# A Comparative Evaluation of Meta-Learning Models for Few-Shot Chest X-Ray Disease Classification

**DOI:** 10.3390/diagnostics15182404

**Published:** 2025-09-21

**Authors:** Luis-Carlos Quiñonez-Baca, Graciela Ramirez-Alonso, Fernando Gaxiola, Alain Manzo-Martinez, Raymundo Cornejo, David R. Lopez-Flores

**Affiliations:** 1 Computer Vision and Data Science Lab, Facultad de Ingeniería, Universidad Autónoma de Chihuahua, Circuito Universitario Campus II, Chihuahua 31125, Mexico; p232278@uach.mx; 2 Facultad de Ingeniería, Universidad Autónoma de Chihuahua, Circuito Universitario Campus II, Chihuahua 31125, Mexico; lgaxiola@uach.mx (F.G.); amanzo@uach.mx (A.M.-M.); rcornejo@uach.mx (R.C.); 3 División de Estudios de Posgrado e Investigación, Tecnológico Nacional de México/IT de Chihuahua, Ave. Tecnológico 2909, Chihuahua 31200, Mexico; david.lf@chihuahua.tecnm.mx

**Keywords:** meta-learning, few-shot learning, chest X-ray, disease classification

## Abstract

**Background/Objectives**: The limited availability of labeled data, particularly in the medical domain, poses a significant challenge for training accurate diagnostic models. While deep learning techniques have demonstrated notable efficacy in image-based tasks, they require large annotated datasets. In data-scarce scenarios—especially involving rare diseases—their performance deteriorates significantly. Meta-learning offers a promising alternative by enabling models to adapt quickly to new tasks using prior knowledge and only a few labeled examples. This study aims to evaluate the effectiveness of representative meta-learning models for thoracic disease classification in chest X-rays. **Methods**: We conduct a comparative evaluation of four meta-learning models: Prototypical Networks, Relation Networks, MAML, and FoMAML. First, we assess five backbone architectures (ConvNeXt, DenseNet-121, ResNet-50, MobileNetV2, and ViT) using a Prototypical Network. The best-performing backbone is then used across all meta-learning models for fair comparison. Experiments are performed on the ChestX-ray14 dataset under a 2-way setting with multiple *k*-shot configurations. **Results**: Prototypical Networks combined with DenseNet-121 achieved the best performance, with a recall of 68.1%, an F1-score of 67.4%, and a precision of 0.693 in the 2-way, 10-shot configuration. In a disease-specific analysis, Hernia obtains the best classification results. Furthermore, Prototypical and Relation Networks demonstrate significantly higher computational efficiency, requiring fewer FLOPs and shorter execution times than MAML and FoMAML. **Conclusions**: Prototype-based meta-learning, particularly with DenseNet-121, proves to be a robust and computationally efficient approach for few-shot chest X-ray disease classification. These findings highlight its potential for real-world clinical applications, especially in scenarios with limited annotated medical data.

## 1. Introduction

Deep learning models have demonstrated exceptional performance in image-based disease classification [1]. However, these techniques require substantial amounts of labeled data for training. In the medical domain, obtaining labeled data can be time-consuming and costly, especially for rare or emerging diseases with low prevalence. Additionally, the labeling process often demands specialized expertise and equipment, which may not always be available, making data annotation challenging or even impossible in certain contexts [2]. Drawing inspiration from human learning processes, meta-learning has emerged as a promising solution, enabling models to “learn to learn” from various tasks and swiftly adapt to new tasks with minimal data examples [3,4,5,6]. Meta-learning can be divided into two stages: meta-training and meta-testing. During the meta-training phase, the model is trained on a set of known classes, enabling it to learn higher-level parameters that enhance its ability to generalize across tasks. In the meta-testing phase, the model is evaluated on new tasks using previously unseen data, assessing its capacity to adapt and perform effectively with minimal training examples. This ability is particularly advantageous in medical imaging, where acquiring large-scale annotated datasets is challenging. In chest X-ray disease classification, meta-learning enables models to quickly adapt to new conditions or rare diseases by using prior knowledge from related diagnostic tasks.

Vettoruzzo et al. [7] categorize meta-learning into three main types: black-box meta-learning methods, optimization-based meta-learning methods, and metric-based meta-learning methods. Black-box meta-learning methods, also known as model-based meta-learning methods, incorporate memory components that store knowledge from previous tasks, enabling rapid adaptation to new tasks. Examples of models in this category include Memory-Augmented Neural Network (MANN) [8] and Simple Neural Attentive Learner (SNAIL) [9]. Optimization-based meta-learning methods approach the problem as a bi-level optimization process, consisting of an inner loop and an outer loop. In the inner loop, the model optimizes task-specific parameters typically from the support set, while, in the outer loop, the initial set of meta-parameters is updated to minimize the meta-loss, which is computed by evaluating the adapted model on a separate query set across multiple tasks. The most representative models are Model-Agnostic Meta-Learning (MAML) [10] and Meta-SGD [11]. Metric-based meta-learning models address the classification problem by assessing the similarity or dissimilarity between new instances and support examples within a learned representation space. These models project data points into a common feature space, enabling them to capture relationships across multiple classes. The representative models are Prototypical Networks [12], Matching Networks [13], and Relation Networks [14].

Given that the effectiveness of meta-learning models is tightly coupled with the quality of feature representations, the choice of backbone architecture, which is responsible for the feature extraction process, plays a critical role in downstream performance. The main objectives of feature extraction include transforming raw data to reduce redundancy, enhancing discriminative representations, and capturing spatial structures relevant to the target task [15]. In meta-learning, these extractors serve as backbones that generate meaningful representations for few-shot classification. Commonly used backbones include ResNet [16], DenseNet [17], MobileNetV2 [18], and Vision Transformers (ViTs) [19]. ConvNeXt is a recent deep learning architecture introduced by Liu et al. [20], designed to modernize conventional Convolutional Neural Networks, CNNs, by incorporating architectural enhancements inspired by vision transformers while retaining the efficiency and simplicity of standard convolutions. ConvNeXt has shown promising results in chest X-ray disease classification by effectively capturing local image features and providing strong representational capacity [21,22,23]. However, its integration into meta-learning pipelines—particularly under few-shot conditions—remains underexplored.

Building on this motivation, our study offers several novel contributions to the field of few-shot medical image classification.

- We perform a comprehensive evaluation of five backbone architectures—ConvNeXt, DenseNet-121, ResNet-50, MobileNetV2, and Vision Transformers (ViT)—to determine their effectiveness and their impact on classification accuracy, representation quality, and generalization in few-shot chest X-ray tasks.
- Under a unified protocol, we compare the performance and computational efficiency of four representative meta-learning approaches: the metric-based Prototypical Networks and Relation Networks, and the optimization-based MAML and FoMAML.
- We demonstrate that the combination of Prototypical Networks with DenseNet-121 delivers the best results, achieving 68.1% recall in a 2-way, 10-shot configuration, and emphasize its suitability for deployment in medical scenarios with limited labeled data.

The rest of this paper is organized as follows: Section 2 reviews related work. Section 3 outlines the proposed methodology. Section 4 describes the experimental setup, and Section 5 presents the results. Section 6 provides a discussion of the findings, and, finally, Section 7 concludes this paper.

## 2. Related Work

Meta-learning has gained increasing attention in medical imaging as a promising approach to address data scarcity in diagnostic tasks. This section provides an overview of recent studies that apply meta-learning methods to classification problems involving chest X-rays, CT scans, and other imaging modalities, summarizing their methodological strategies.

As previously noted, meta-learning techniques can be categorized into three groups: black-box methods, optimization-based methods, and metric-based methods. These approaches have been successfully applied to various medical image classification tasks, including disease diagnosis using chest X-ray (CXR), computed tomography (CT) scans, or other image modalities. For instance, within the optimization-based category, Ouahab, Ben-Ahmed, and Fernandez-Maloigne [24] proposed a MAML-based framework that incorporates an attention mechanism and a vanilla CNN backbone to transfer knowledge from common to low-prevalence diseases. The model was evaluated on low-prevalence skin and thorax disease datasets employing a two-stage process—meta-training and meta-testing—to learn transferable representations from base disease classes and adapt efficiently to rare disease conditions. The authors reported an average AUC of 84.3% for skin diseases and 73.4% for thoracic diseases under a 2-way, 5-shot classification setting. Extending this paradigm, Singh et al. [25] introduced MetaMed, a meta-learning framework that integrates advanced data augmentation strategies, such as MixUp, CutOut, and CutMix, during the meta-training phase. MetaMed, constructed as a gradient-based model, was evaluated on diverse medical imaging datasets, including histopathological images (BreakHis) [26], dermoscopic skin lesion images (ISIC 2018) [27], and cytology images (Pap Smear) [28]. The best reported results achieved an average accuracy of 82.75% on the BreakHis dataset, 84.25% on the ISIC 2018 dataset, and 93.37% on the Pap Smear dataset under a 2-way, 10-shot configuration. Following a MAML-based framework with distinct inner-loop (local) and outer-loop (global) updates, Chen et al. [29] proposed a meta-learning model for pneumonia detection that incorporates hierarchical relationships among diseases. Their approach uses a disease hierarchy tree based on the International Classification of Diseases (ICD-11) to guide a hierarchical classification module capable of multi-level disease discrimination. The model employs a ResNet25 backbone pre-trained on ImageNet and is evaluated using a combined dataset constructed from the CoronaHack-Chest X-ray and COVID-Chest X-ray datasets [30]. The authors reported an average classification accuracy of 85.77% ± 0.67 under a 2-way, 5-shot configuration.

In contrast to optimization-based approaches, metric-based meta-learning methods classify query samples by comparing them to class prototypes or reference embeddings within a learned feature space, using either predefined or learned distance metrics. As highlighted in [3], these methods offer two key advantages: they are conceptually simple, facilitating implementation and interpretability, and they enable efficient inference, especially in scenarios with a limited number of classes and samples. In contrast to black-box or optimization-based methods, metric-based approaches avoid complex gradient-based adaptation at test time. Among these, Prototypical Networks [12] have gained widespread adoption in few-shot image classification due to their simplicity and competitive performance. For instance, Anandhi et al. [31] proposed Protonet++, a framework for lung disease diagnosis that integrates a U-Net segmentation module between image preprocessing and classification. The pipeline begins with Contrast Limited Adaptive Histogram Equalization (CLAHE) to enhance image contrast, followed by U-Net-based segmentation to isolate relevant anatomical regions, and concludes with a prototypical network for classifying diseases such as lung cancer, tuberculosis, and pneumonia. The authors evaluated their framework using the Tuberculosis Chest X-ray Database, the Kaggle Chest X-ray dataset for pneumonia, and the JSRT dataset for lung cancer, reporting a maximum accuracy of 97.51%. However, they did not specify how these datasets were combined to form the meta-train and meta-test splits, nor did they provide details on the number of *n*-ways or *k*-shots used in their few-shot configuration.

Building on contrastive principles, Chen et al. [32] developed a few-shot learning framework for COVID-19 diagnosis using chest CT scans. Their method combines stochastic data augmentation and contrastive learning to train a feature encoder, which is subsequently used within a prototypical network for classification. The framework was validated on two datasets, including the COVID-19 CT dataset and one provided by the Italian Society of Medical and Interventional Radiology (MegSeg). The authors combined the two datasets into a single cohort, and, to prevent overlap, the support and query sets were divided at the patient level. The best performance was obtained under a 2-way, 5-shot setting, with an AUC of 0.946 ± 0.010. Similarly, Shorfuzzaman et al. [33] introduced MetaCOVID, a metric-based model that employs Siamese networks with a VGG-16 backbone. Their training strategy combines binary cross-entropy and contrastive loss to distinguish COVID-19 and pneumonia cases from chest X-rays. The authors created a balanced dataset by combining COVID-19, non-COVID pneumonia, and normal chest X-ray images from public repositories. MetaCOVID achieved its best performance under a 3-way, 10-shot setting with 95.6% accuracy. More recently, Ouahab and Ahmed [34] proposed ProtoMed, a lightweight meta-learning model based on a Prototypical Network with an auxiliary branch for regularization. This auxiliary branch incorporates a supervised contrastive loss that encourages intra-class compactness and inter-class separability in the embedding space. Their approach was evaluated independently on three benchmark datasets: ISIC 2018 for skin lesions, BreakHis for breast cancer histopathology, and NIH Chest X-ray14 (augmented with COVID-19 cases) for thoracic diseases. The best results were obtained under the 2-way 10-shot configuration, achieving 84.61% accuracy on ISIC 2018, 84.31% accuracy on BreakHis, and 78.36% accuracy on Chest X-ray14 augmented with COVID-19 cases.

On the other hand, Paul et al. [35] proposed a model composed of a coarse-learner module and a saliency-based classifier. The coarse-learner employs a DenseNet architecture to extract common features from chest X-ray images. The saliency-based classifier consists of 15 autoencoders, each mapping the input to a latent feature space, followed by a decoder that reconstructs the input from this representation. Their framework was evaluated on the NIH ChestX-ray14 dataset, with additional testing on the Open-i dataset considering a 3-way, 5-shot configuration. The authors explored different setups for base and novel classes, reporting in one configuration F1-scores of 0.63 ± 0.04, 0.38 ± 0.02, and 0.49 ± 0.02 for fibrosis, hernia, and pneumonia, respectively.

Meta-learning has also been explored under unsupervised settings, where labeled data is scarce or unavailable [36]. In this paradigm, known as Unsupervised Meta-Learning (UML), the model learns from unlabeled data by generating pseudo-tasks through clustering, data augmentation, or pseudo-labeling strategies. These synthetic tasks simulate the few-shot learning setting, allowing the model to acquire transferable knowledge without relying on manual annotations. Zheng et al. [37] proposed a metric-based UML approach that leverages relation networks to capture and memorize relationships among medical images. Additionally, they introduced a self-knowledge distillation mechanism, enabling the model to refine its internal representations through its own predictions. Their framework employs DenseNet-121 as the feature extractor and is trained on a custom dataset combining chest X-ray (CXR) and CT images for COVID-19 and pneumonia diagnosis. Although their proposal follows a *n*-way, *k*-shot formulation, the tasks are constructed artificially and differ from conventional supervised settings. In their case, performance was reported using global metrics rather than per-task accuracy, achieving up to 99.5% accuracy on X-rays and 98.6% accuracy on CT scans.

In a similar vein with UML and metric-based approach, Ding Lv et al. [38] developed a framework that combines multiple similarity measures with a Mini-Batch Sampling (MBS) strategy to efficiently simulate few-shot tasks from unlabeled data. Their method constructs episodic tasks by randomly dividing augmented samples into support and query sets within a single forward pass. Using ResNet-12 as backbone, the model was evaluated independently on three reconstructed medical datasets—BLOOD (cell images), PATHOLOGY (histopathology), and CHEST (radiographs). The best results were achieved under a 3-way, 10-shot setting, with 75.91% accuracy on BLOOD, 86.87% on PATHOLOGY, and 52.23% on CHEST.

Despite the growing interest in applying meta-learning to medical image classification, prior studies have mainly focused on optimization-based methods [24,25,29] and metric-based models [31,32,33,34,35], considering, in some cases, unsupervised settings [37,38]. In contrast, to the best of our knowledge, black-box strategies have not been explored in this context, possibly due to their perceived lack of interpretability, a critical aspect in medical applications. Nevertheless, existing optimization- and metric-based studies have paid limited attention to the impact of using different feature representations in few-shot classification tasks. Furthermore, the integration of modern backbone architectures is still underexplored, with most analyses focused on a narrow range of thoracic conditions.

Motivated by these limitations, this study conducts a comprehensive evaluation of convolutional backbone architectures for few-shot chest X-ray classification, incorporating contrast-enhanced preprocessing. We begin by identifying the most effective backbone using a Prototypical Network, a widely adopted metric-based model. The selected backbone is then used within a Relation Network to assess consistency across metric-based approaches. Finally, we implement two representative optimization-based models—Model-Agnostic Meta-Learning (MAML) and its first-order variant (FoMAML)—and analyze their performance under *2*-way and various *k*-shot configurations.

## 3. Methodology

In few-shot learning, meta-tasks are constructed following an *n*-way, *k*-shot scheme, where each meta-task consists of *k* samples per class for a total of *n* classes. Typically, *k* is set to a small value (e.g., 1 or 5), while *n* ranges between 2 and 10 [39]. The overall process is organized into two main stages: meta-training and meta-testing [40]. During meta-training, the model learns from a set of meta-tasks sampled from common disease classes, with both support and query examples drawn from the meta-training set. In the meta-testing stage, the model is evaluated on meta-tasks sampled from previously unseen disease classes, assessing its ability to adapt to novel conditions with limited data. Importantly, the disease classes in the meta-training and meta-testing sets are mutually exclusive to ensure proper generalization.

Formally, we define $D_{\mathrm{tr}}$ and $D_{\mathrm{ts}}$ as the meta-training and meta-testing datasets, which contain base and novel classes, respectively. Additionally, we consider a meta-task distribution P(τ), from which meta-tasks are sampled during both stages. Each meta-task $\tau_{i}$ consists of a support set containing N×K samples and a query set containing N×Q samples, where *Q* is the number of query samples per class. The support and query sets are disjoint.

For each meta-task $\tau_{i}$, we denote the support set as $D_{i}^{S}$ and the query set as $D_{i}^{Q}$, containing N×K and N×Q samples, respectively. The objective of meta-learning is to learn model parameters θ that minimize the expected loss over the meta-task distribution.

**Figure 1:**
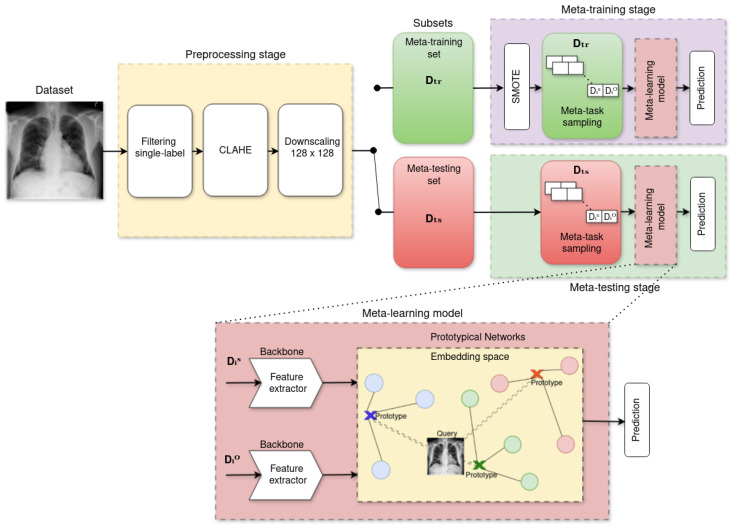
Methodological pipeline for evaluating backbone architectures within a Prototypical Network-based meta-learning framework.

The overall workflow for identifying the most effective backbone (or feature extractor) architecture, using a Prototypical Network as the baseline metric-based model, is illustrated in Figure 1. The process initiates with a preprocessing stage that applies Contrast Limited Adaptive Histogram Equalization (CLAHE) to enhance image contrast, followed by downscaling to standardize resolution and reduce computational cost. The meta-learning pipeline is then divided into meta-training and meta-testing stages, each structured around episodic tasks composed of a support and a query set. To mitigate class imbalance during meta-training, the Synthetic Minority Over-sampling Technique (SMOTE) is applied exclusively to the support sets. This step is deliberately excluded during meta-testing to preserve the natural class distribution and avoid bias when assessing model generalization on novel disease classes.

### 3.1. Preprocessing

**Figure 2:**
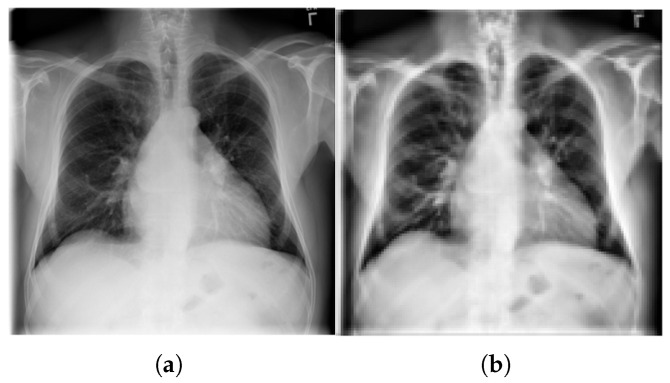
Comparison between (**a**) the original grayscale chest X-ray image at a resolution of 1024×1024 pixels, and (**b**) the image after preprocessing using CLAHE and rescaling to 128×128 pixels.

The preprocessing stage consists of three main steps. First, we prepare the ChestX-ray14 dataset [41], which contains 112,120 chest X-ray images annotated with 14 thoracic disease classes and captured at varying resolutions. To align with the single-label classification setting, we filter the dataset to include only images annotated with a single disease and captured in either posteroanterior (PA) or anteroposterior (AP) views, yielding a refined subset of 30,963 images. Second, we enhance the visual quality of the images by applying Contrast Limited Adaptive Histogram Equalization (CLAHE), which improves local contrast and highlights disease-relevant patterns. Finally, all images are resized to 128×128 pixels to reduce computational cost and ensure consistent input dimensions across models. Figure 2 illustrates the effect of CLAHE enhancement followed by image resizing.

### 3.2. Meta-Training

The meta-training stage is performed exclusively on the meta-training dataset $D_{\mathrm{tr}}$, which includes the 11 most frequent disease classes, referred to as common diseases, from the ChestX-ray14 dataset: Atelectasis, Cardiomegaly, Consolidation, Effusion, Emphysema, Fibrosis, Infiltration, Mass, Nodule, Pleural Thickening, and Pneumothorax. We adopt an *n*-way *k*-shot few-shot classification scheme, where *n* disease classes are randomly selected for each meta-task, and *k* support images and *Q* query images are sampled per class. It is ensured that the support and query sets are disjoint within each meta-task. Each meta-task is constructed dynamically by sampling the support and query sets from $D_{\mathrm{tr}}$. Additionally, episodic sampling was employed to promote diversity and prevent overfitting to specific disease classes. The samples are then processed through the feature extractor. Subsequently, classification is performed using a meta-learning model.

The objective of the meta-training stage is to enable the model to learn transferable representations and decision boundaries from the common disease classes, allowing it to rapidly adapt to novel, unseen disease classes during meta-testing with only a few labeled examples.

### 3.3. Meta-Testing

**Figure 3:**
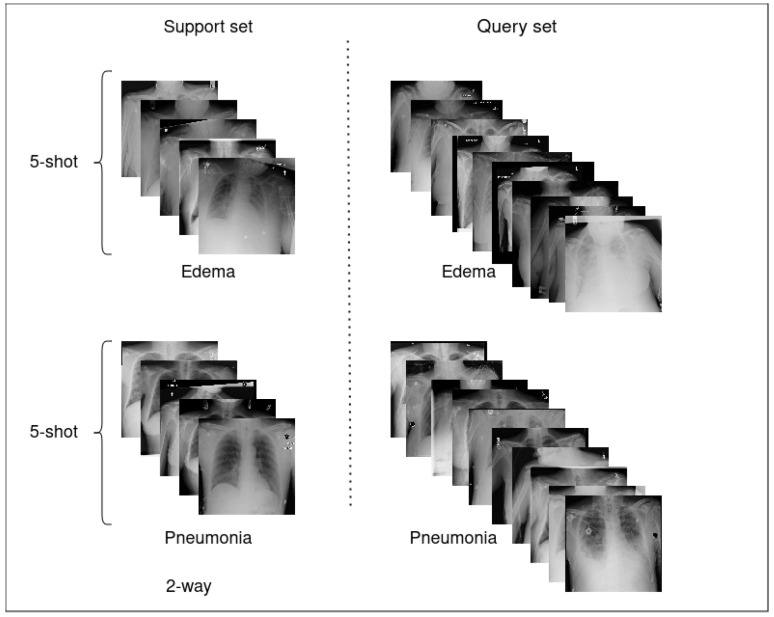
Illustration of a 2-way, 5-shot learning meta-task involving the disease classes Edema and Pneumonia, randomly selected from the set of three novel classes. The support set includes five labeled samples per class, while the query set comprises ten samples per class.

The meta-testing phase evaluates the model’s ability to generalize to previously unseen disease classes. Specifically, the three least frequent conditions in the ChestX-ray14 dataset—Pneumonia, Hernia, and Edema—are designated as novel classes for this stage. This selection aims to reflect real-world clinical scenarios where data scarcity is common for certain conditions. To ensure consistency, meta-tasks during meta-testing are constructed using the same *n*-way *k*-shot setting employed in meta-training, with disjoint support and query sets (see Figure 3). The meta-learning models are evaluated using the parameters learned during meta-training, without any additional fine-tuning, thereby directly testing their capacity for fast adaptation. Model performance is assessed using three standard metrics: recall, precision, and F1-score. These metrics offer a comprehensive evaluation of the model’s ability to classify query samples, with particular emphasis on sensitivity and discriminative capability, two critical aspects in the medical diagnosis context.

### 3.4. Backbones (Feature Extractors)

This study evaluates and compares the performance of five neural architectures as feature extractors, encompassing both lightweight and high-capacity models, all pretrained on ImageNet. These backbones are employed to generate fixed feature embeddings from chest X-ray images, which are subsequently used as input to the meta-learning models. For integration into the meta-learning framework, the final classification layer is removed, and the output of the last hidden layer is extracted as a fixed feature representation. The five evaluated backbone architectures are described below.

#### 3.4.1. DenseNet-121

The DenseNet-121 architecture, introduced by Huang et al. [17], comprises four dense blocks with 6, 12, 24, and 16 densely connected convolutional layers, respectively. Each layer applies a 1×1 convolution followed by a 3×3 convolution, both integrated with Batch Normalization (BN) and ReLU, and its output is concatenated with all preceding feature maps within the block. This dense connectivity promotes feature reuse, enhances information flow, and mitigates vanishing gradients. Between blocks, transition layers with a 1×1 convolution and 2×2 average pooling reduce spatial resolution and feature map size. The network concludes with a global 7×7 average pooling before classification, enabling stable and efficient training through improved feature propagation.

#### 3.4.2. ResNet-50

ResNet-50, proposed by He et al. [16], addresses the vanishing gradient problem through residual connections that preserve gradient flow by learning identity mappings. The architecture is organized into five stages: an initial 7×7 convolution with stride two and a 3×3 max pooling, followed by 3, 4, 6, and 3 residual blocks. Each block adopts a bottleneck design of three layers—1×1 for dimensionality reduction, 3×3 for processing, and 1×1 for dimensionality restoration—enabling efficient training and strong performance in large-scale image classification.

#### 3.4.3. MobileNetV2

MobileNetV2, proposed by Sandler et al. [18], is designed for efficient deployment on resource-constrained devices. It builds on depthwise separable convolutions by introducing inverted residual connections and linear bottlenecks, which enhance information flow and reduce computational cost. The architecture includes 17 inverted residual blocks between an initial convolution and the final classification layer, where each block expands the input, applies a depthwise convolution, and projects back to lower dimensions through a linear layer, preserving information for efficient training.

#### 3.4.4. ViT-Base

Dosovitskiy et al. [19] introduced the Vision Transformer (ViT), which adapts the Transformer architecture from NLP to image classification. Images are split into fixed-size patches, linearly embedded as tokens, enriched with positional encodings, and processed by a Transformer encoder, followed by an MLP head for classification. The ViT-Base model used here has 12 encoder layers, 12 attention heads, and a hidden size of 768.

#### 3.4.5. ConvNeXt

In this study, we employ the ConvNeXt-Tiny variant [20], pretrained on ImageNet, for feature extraction. The architecture consists of four hierarchical stages with ConvNeXt blocks inspired by Transformers, each including a 7×7 depthwise convolution, Layer Normalization, a 1×1 pointwise expansion, GeLU activation, and a 1×1 projection, all connected through residual links. The Tiny configuration is defined by B=(3,3,9,3) blocks and C=(96,192,384,768) channels, enabling progressive receptive field expansion and multi-scale representation learning.

### 3.5. Prototypical Networks

Prototypical Networks [12] are a class of metric-based meta-learning models designed for few-shot classification. They operate by learning an embedding space in which classification is performed based on the distance between query samples and prototype representations of each class. A prototype is defined as the mean vector of the embedded support examples corresponding to a given class, serving as a representative point in the feature space.

In an *N*-way *K*-shot classification task, the prototype of class *n* in episode $\tau_{i}$ is calculated as follows:

(1) $$c_{n}=\frac{1}{K}\sum_{\left(x_{j},y_{j}\right)\in D_{i}^{S}}f_{\theta }\left(x_{j}\right)\cdot 1\left\{y_{j}=n\right\},$$

where $c_{n}$ denotes the prototype of class *n*, $D_{i}^{S}$ is the support set of the *i*-th meta-task $\tau_{i}$, $f_{\theta }$ is the embedding function parameterized by θ, and $1\{y_{j}=n\}$ is the indicator function that equals 1 if $y_{j}=n$, and 0 otherwise.

During meta-testing, each query sample $x_{q}\in D_{i}^{Q}$ is classified according to its distance to the prototypes. The probability of assigning $x_{q}$ to class *n* is computed as follows:

(2) $$p_{\theta }\left(y_{q}=n\;|\;x_{q}\right)=\frac{\exp(-d\left(f_{\theta }\left(x_{q}\right),c_{n}\right))}{\sum_{n^{\prime }=1}^{N}\exp\left(-d\left(f_{\theta }\left(x_{q}\right),c_{n^{\prime }}\right)\right)},$$

where d(·,·) denotes a distance metric, typically the Euclidean distance or cosine similarity.

The model is trained by minimizing the cross-entropy loss over the query set $D_{i}^{Q}$ of each meta-task:

(3) $$L=-\sum_{\left(x_{q},y_{q}\right)\in D_{i}^{Q}}\log p_{\theta }\left(y_{q}\;|\;x_{q}\right).$$

### 3.6. Relation Networks

Relation Networks [14] are a class of metric-based meta-learning models designed to perform few-shot classification by learning to compare samples based on their pairwise relationships. In contrast to Prototypical Networks, which compute distances to class prototypes in a fixed embedding space, Relational Networks employ a learnable relation module that estimates similarity scores between support and query examples.

Given a support set $D_{i}^{S}$ and a query sample $x_{q}\in D_{i}^{Q}$ in meta-task $\tau_{i}$, each support image $x_{j}$ is first embedded using a shared feature extractor $f_{\theta }$. The relation network $g_{\phi }$ then takes the concatenation of the query and support embeddings as input and outputs a similarity score:

(4) $$r_{j,q}=g_{\phi }\left([f_{\theta }\left(x_{j}\right),f_{\theta }\left(x_{q}\right)]\right),$$

where [·,·] denotes the concatenation operation, and $r_{j,q}$ is the relation score between query $x_{q}$ and support example $x_{j}$.

In our implementation, the relation module $g_{\phi }$ is instantiated as a Multilayer Perceptron (MLP) that takes as input the output of the feature extractor $f_{\theta }$. The MLP is a feedforward neural network whose input layer receives the concatenated embeddings of the support and query samples, resulting in an input dimension of 2× feature dimension. This is followed by two hidden layers: the first reduces the dimensionality to 512, followed by Batch Normalization, a ReLU activation function, and a Dropout layer with a rate of 0.3. The second hidden layer further reduces the dimension to 256, also followed by Batch Normalization, ReLU, and Dropout (0.3). Finally, the output layer reduces the dimension from 256 to 1, producing a relation score that quantifies the similarity between each query–support pair.

In *n*-way *k*-shot tasks, the model aggregates relation scores across all *k* examples per class and predicts the label of $x_{q}$ based on the class with the highest average relation score. The model is trained by minimizing the cross-entropy loss between the predicted relation scores and the ground-truth labels (encoded as one-hot vectors).

### 3.7. Model-Agnostic Meta-Learning (MAML) and First-Order MAML (FoMAML)

Model-Agnostic Meta-Learning (MAML) [10] is a gradient-based meta-learning framework that aims to find a model initialization capable of rapid adaptation to new tasks using only a small number of gradient updates and limited data. Unlike distance-based methods that rely on fixed embedding spaces, MAML focuses on learning a set of parameters θ that serve as a good starting point for fine-tuning on novel tasks.

The learning process consists of two nested optimization loops. In the inner loop, the model adapts its parameters to a specific task $\tau_{i}$ by performing one or more gradient descent steps using the task-specific loss $L_{\tau_{i}}$:

(5) $$\theta_{i}^{\prime }=\theta -\alpha \nabla_{\theta }L_{\tau_{i}}\left(f_{\theta }\right),$$

where α is the inner learning rate, and $\theta_{i}^{\prime }$ denotes the adapted parameters for task $\tau_{i}$.

In the outer loop, the initial parameters θ are updated based on the performance of the adapted model $\theta_{i}^{\prime }$ on the corresponding query set:

(6) $$\theta \leftarrow \theta -\beta \nabla_{\theta }\sum_{\tau_{i}∼p\left(\tau \right)}L_{\tau_{i}}\left(f_{\theta_{i}^{\prime }}\right),$$

where β is the meta learning rate, and p(τ) denotes the task distribution.

First-Order MAML (FoMAML) simplifies this procedure by omitting the second-order derivative term when computing the meta-gradient, which substantially reduces computational overhead while achieving similar performance in practice. Both MAML and FoMAML are compatible with any differentiable model and training objective, making them broadly applicable in few-shot learning contexts.

## 4. Experiments

This section presents the experimental protocol, covering dataset preprocessing and class partitioning tailored for few-shot learning. We then describe the meta-training and meta-testing procedures, emphasizing the episodic task construction and model configurations used to assess generalization to novel disease classes.

### 4.1. Dataset Description

We evaluate the proposed methodology using the ChestX-ray14 dataset [41] released by the National Institutes of Health. This dataset contains 112,120 frontal chest X-ray images from 30,805 unique patients, annotated with 14 thoracic disease categories. For our experiments, we construct a subset of 30,963 images that are strictly single-labeled and acquired in posteroanterior (PA) and anteroposterior (AP) views, ensuring consistency and diagnostic clarity. The distribution of images across the 14 disease categories is as follows: Infiltration (9547), Atelectasis (4215), Effusion (3955), Nodule (2705), Pneumothorax (2194), Mass (2139), Consolidation (1310), Pleural Thickening (1126), Cardiomegaly (1093), Emphysema (892), Fibrosis (727), Edema (628), Pneumonia (322), and Hernia (110). For meta-learning purposes, the dataset is divided into 11 base classes for meta-training and 3 novel classes for meta-testing. The three least frequent conditions—Pneumonia, Hernia, and Edema—are selected as novel classes to simulate low-resource diagnostic scenarios. All images are rescaled to 128×128 pixels to reduce computational cost.

### 4.2. Experimental Setup

The proposed methodology was implemented using PyTorch version 2.5.1 and trained on a workstation equipped with an NVIDIA GeForce RTX 2080 GPU, an Intel Core i9 processor running at 3.7 GHz, and 32 GB of RAM. Training was performed on the meta-training set, while evaluation was conducted on the novel classes from the meta-testing set.

Meta-training. The model was trained for 1000 episodes using on-the-fly generation of meta-tasks in a 2-way classification setting. To evaluate performance under varying data availability, each episode was conducted under a *k*-shot configuration, with k∈{5,7,10}, corresponding to 5, 7, or 10 support samples per class. Image features were extracted using five distinct backbone architectures pretrained on ImageNet. Each model processed grayscale chest X-ray images that were first enhanced with Contrast Limited Adaptive Histogram Equalization (CLAHE) and subsequently resized to 128×128 pixels.

The meta-training set included a total of 29,903 single-label chest X-ray images from the base disease classes (see Table 1).

SMOTE. Given the class imbalance present in the meta-training set, the Synthetic Minority Over-sampling Technique (SMOTE) [42] was applied to reduce prediction bias during meta-task construction. Previous studies have demonstrated the effectiveness of SMOTE in medical imaging applications [43,44,45], including COVID-19 and pneumonia detection in chest X-rays, where performance improvements were reported when compared to models trained on imbalanced data. In the ChestX-ray14 dataset, the majority class corresponds to Infiltration with 9547 images. As a result, 75,114 synthetic samples were generated to balance the remaining classes, creating a more equitable pool for support and query selection. The number of synthetic samples added per class was as follows: Cardiomegaly (8454), Nodule (6842), Emphysema (8655), Effusion (5592), Atelectasis (5332), Mass (7408), Pneumothorax (7353), Pleural Thickening (8421), Fibrosis (8820), and Consolidation (8237); no augmentation was required for Infiltration.

Meta-testing. During the meta-testing stage, the model was evaluated exclusively on novel disease classes using the parameters learned during meta-training. Evaluation was performed under the same 2-way classification setting, with *k*-shot configurations where k∈5,7,10. Each episode included ten query samples per class and was repeated over a total of 1000 episodes. Chest X-ray images in the meta-test set were preprocessed using the same pipeline as in meta-training—enhancement with CLAHE followed by resizing to 128×128 pixels—to ensure consistency in visual features.

Table 1 summarizes the distribution of images for both base classes (used during meta-training) and novel classes (used exclusively during meta-testing).

## 5. Results

This section presents the experimental results, with the first analysis addressing the evaluation of a Prototypical Network combined with five different backbone architectures: DenseNet-121, ResNet-50, MobileNetV2, Vision Transformer (ViT) Base, and ConvNeXt-Tiny. Each configuration was assessed under a 2-way, *k*-shot classification setting, with *k* values of 5, 7, and 10. We then compare the performance and computational efficiency of four meta-learning algorithms—Model-Agnostic Meta-Learning (MAML), First-Order MAML (FoMAML), Relation Networks, and Prototypical Networks—using the best backbone architecture. This choice ensures a consistent and fair comparison. After, we provide a breakdown of results per disease to offer a more detailed analysis of model performance across specific conditions. All implemented meta-learning models follow a consistent meta-learning framework, comprising 1000 meta-training episodes and 1000 meta-testing episodes. Cross-entropy loss is used as the objective function throughout. For MAML, we employ the Lion optimizer with a learning rate of 0.01 in the outer loop, while the inner loop uses Lion with a learning rate of $1\times 10^{-4}$.

### 5.1. Backbone Evaluation

In medical diagnostics, recall (also known as sensitivity) is a critical performance metric, as false negatives can lead to severe consequences, including delayed treatment or mortality [46]. According to Hicks et al. [47], this metric is the most important in medical studies, where minimizing missed positive cases is paramount; accordingly, it directly reflects a model’s ability to identify patients with the target condition. Consequently, we chose recall as the primary metric to evaluate different backbone architectures in a few-shot learning context and to determine the optimal number of shots for our experiments. Our evaluation methodology involves 2-way meta-task episodes. Within each episode, we compute the recall for each class and then calculate the macro-averaged recall. Since our episodes are class-balanced (i.e., they contain the same number of support and query samples per class), the macro-recall is mathematically equivalent to the accuracy for each meta-task. The final reported scores are the average over 1000 such episodes, and 10 independent runs. Table 2 summarizes the recall scores (%) for the different backbones under 5-shot, 7-shot, and 10-shot settings. The results indicate that the DenseNet-121 architecture provided the best performance, achieving a peak recall of 68.1% ± 1.7% in the 10-shot configuration. Therefore, it was selected as the optimal backbone for our meta-learning model.

**Figure 4:**
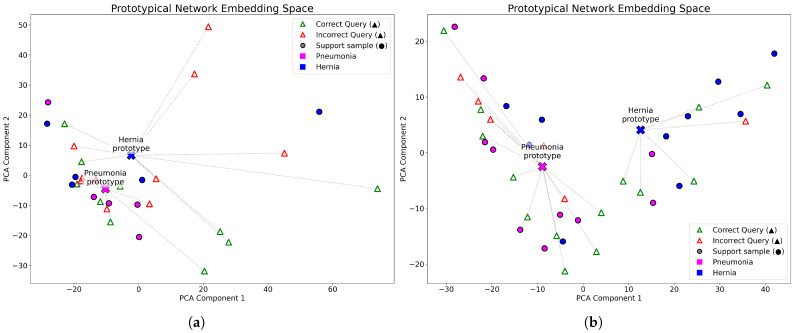
Comparison of 2D PCA embeddings generated by a Prototypical Network with DenseNet121 backbone. (**a**) 2-way, 5-shot; (**b**) 2-way, 10-shot episode.

To further explore how the number of support examples influences the learned representations, Figure 4 compares the embedding spaces generated with a DenseNet-121 backbone under 5-shot and 10-shot configurations. Each 2-way meta-test episode includes 10 query images per class, and the high-dimensional embeddings were projected into two dimensions using PCA. The visualizations correspond to Pneumonia and Hernia classification tasks. In the 10-shot configuration (right), the class prototypes are more distinctly separated compared to the 5-shot scenario, suggesting that a higher number of support examples yields more compact and discriminative clusters, thereby improving query classification.

Based on the insights gained from Figure 4 and the results presented in Table 2, the 10-shot configuration was selected for a more detailed evaluation of backbone performance across additional metrics. Table 3 presents the results under a 2-way, 10-shot classification setup, focusing on the novel disease classes Pneumonia, Hernia, and Edema. The findings further underscore the importance of backbone selection in few-shot learning. To evaluate whether the observed performance differences among the backbone models are statistically significant, we apply the one-sided Wilcoxon signed-rank test with a significance level of α=0.05. This non-parametric test is appropriate for paired data, as all backbones are tested under identical conditions using the same dataset, novel classes, and meta-task configuration. The resulting *p*-values show that DenseNet-121 consistently outperforms MobileNetV2 ($p_{precision}=0.0097$, $p_{recall}=0.0244$, and $p_{F1}=0.0244$), ViT-Base ($p_{precision}=0.0009$, $p_{recall}=0.0009$, and $p_{F1}=0.00097$), and ConvNeXt-T ($p_{precision}=0.0527$, $p_{recall}=0.0419$, and $p_{F1}=0.0419$). In contrast, the comparison with ResNet-50 does not indicate a statistically significant difference ($p_{precision}=0.1875$, $p_{recall}=0.2158$, $p_{F1}=0.1875$). However, DenseNet-121 shows slightly higher average scores across all runs and requires substantially fewer parameters than ResNet-50 (6.95M vs. 23.50M), which reduces the risk of overfitting and lowers computational cost. Therefore, DenseNet-121 is adopted as the backbone network for subsequent analyses.

**Figure 5:**
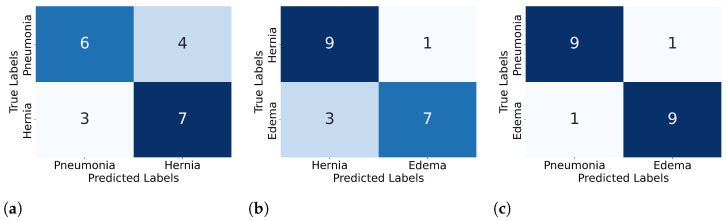
Confusion matrices from Prototypical Networks with a DenseNet121 backbone, showing three 2-way, 10-shot meta-tasks with 10 query samples per class: (**a**) low performance (recall = 0.65), (**b**) moderate (0.80), and (**c**) high (0.90).

Figure 5 presents representative confusion matrices from three selected 2-way, 10-shot meta-test episodes, generated using the Prototypical Network with the DenseNet-121 backbone. Each episode includes 10 query samples per class. Figure 5a–c correspond to episodes with low (recall = 0.65), moderate (0.80), and high (0.90) performance, respectively, showcasing the variability inherent to few-shot learning. These examples are drawn from the 1000 meta-test episodes used to compute the average recall of 68.1% reported for this model.

### 5.2. Meta-Learning Models Evaluation

Considering DenseNet-121 as the backbone architecture, we evaluate four meta-learning models: the previously used Prototypical Network, along with Relation Networks, MAML, and FoMAML. The assessment is conducted under a 2-way classification setting with varying *k*-shot configurations (k∈5,7,10). Table 4 reports the average recall, precision, and F1-score across 1000 episodes for each configuration. As expected, increasing the number of support samples generally improves performance across all metrics. In comparison with the evaluated models, the Prototypical Network consistently yields the best numerical results, highlighting its strength in capturing class-level representations from limited data. To statistically validate these findings, we conduct a one-sided Wilcoxon signed-rank test under the 2-way, 10-shot configuration to compare the four meta-learning models. The resulting *p*-values are all below the α=0.05 threshold, and the pairwise comparisons consistently identify Prototypical Networks as the top-performing approach across all three metrics, significantly outperforming MAML ($p_{precision}=0.0009$, $p_{recall}=0.0009$, and $p_{F1}=0.0009$), FOMAML ($p_{precision}=0.0009$, $p_{recall}=0.0009$, and $p_{F1}=0.0009$), and Relation Networks ($p_{precision}=0.0009$, $p_{recall}=0.0009$, and $p_{F1}=0.0009$). MAML and FOMAML, in turn, are statistically indistinguishable from each other ($p_{precision}=0.8837$, $p_{recall}=0.9033$, and $p_{F1}=0.9472$), but both significantly outperformed Relation Networks.

**Figure 6:**
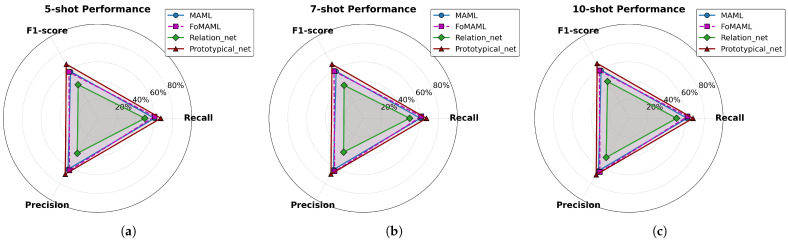
Radar plots comparing the performance of meta-learning models under different *k*-shot settings: (**a**) 5-shot, (**b**) 7-shot, and (**c**) 10-shot. Metrics include Recall, Precision, and F1-score.

To complement these previous results, Figure 6 provides a visual summary of each model’s performance across metrics and shot configurations. This representation highlights the consistent advantage of the Prototypical Network, whose performance metrics trace the outermost boundary in the radar plots (depicted in red), indicating strong generalization capabilities. In contrast, the Relation Network shows the lowest performance across all metrics, with a particularly reduced F1-score, underscoring its difficulty in balancing sensitivity and precision—an essential requirement in medical image classification.

Interestingly, MAML and FoMAML exhibit very similar performance profiles across all *k*-shot settings, with only marginal differences between them. While MAML involves the computation of second-order gradients during meta-training, which increases its computational complexity, FoMAML simplifies the process by relying solely on first-order approximations. As a result, FoMAML achieves comparable performance while reducing training time and resource consumption, positioning it as a more practical option in computationally constrained scenarios.

Table 5 presents a disease-specific evaluation under the 2-way 10-shot configuration. For Pneumonia, MAML achieved the highest recall (34.0 ± 1.4%), precision (0.532 ± 0.010), and F1-score (39.5 ± 1.4%), with FoMAML matching the F1-score (39.5 ± 0.6) and performing closely in the other metrics. Prototypical Network showed a lower recall (28.8 ± 0.6%) but maintained a stable precision (0.502 ± 0.001) and a competitive F1-score (35.7 ± 0.5%), reflecting a conservative prediction strategy that limits false positives. In the Hernia class, MAML achieved the best performance across all three metrics, with the highest recall (46.6 ± 1.6%), precision (0.600 ± 0.011), and F1-score (50.8 ± 1.4%). FoMAML followed closely (46.3 ± 1.0% recall, 0.599 ± 0.006 precision, and 50.6 ± 0.9% F1), demonstrating balanced performance while reducing computational cost compared to MAML by avoiding second-order gradient updates. For Edema, Prototypical Network obtained the highest recall (38.7 ± 2.7%) and the highest F1-score (44.0 ± 2.2%), alongside a solid precision of 0.527 ± 0.011, outperforming all other models in both recall and F1 for this category.

It is important to note that, although MAML and FoMAML deliver strong results—particularly in Pneumonia and Hernia—these gradient-based meta-learning approaches are computationally more demanding during training. MAML requires full second-order optimization, while FoMAML uses first-order approximations, making the latter more efficient but still costlier than metric-based alternatives, such as Prototypical Network, which offer competitive performance with lower training complexity.

Table 6 and Table 7 summarize the computational cost of each evaluated model in terms of floating-point operations (FLOPs) and execution time, respectively. FLOPs were computed using the fvcore library (Licensed under Apache License 2.0. Available at: https://github.com/facebookresearch/fvcore (accessed on 7 July 2025)), with higher values indicating greater computational complexity. Inference FLOPs correspond to the operations required for a complete forward pass per episode, while training FLOPs include both forward and backward passes. FLOPs and execution time per episode were measured under the 2-way, 10-shot setting with 10 query images per class (40 grayscale images of size 128×128), encompassing all meta-learning pipeline steps: task sampling, backbone forward passes, prototype construction and similarity computation (for metric-based methods), and performance calculation. During meta-training, timing also includes loss computation, gradient backpropagation, and optimizer updates, whereas meta-testing accounts for task-specific adaptation and query set evaluation. The “Total FLOPs” and “Total Time” columns report the accumulated values over 1000 episodes, reflecting the overall computational cost for each phase. As expected, gradient-based methods (MAML and FoMAML) incur substantially higher training costs than metric-based approaches (ProtoNet and RelationNet), with FoMAML achieving a marked reduction in training FLOPs while preserving the same inference cost as MAML; meta-testing costs are comparatively closer across methods.

**Figure 7:**
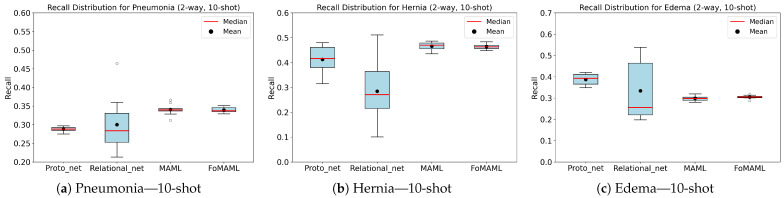
Boxplots of Recall using Prototypical Networks for 10-shot classification of: (**a**) Pneumonia, (**b**) Hernia, and (**c**) Edema.

To evaluate model stability and performance consistency, we analyzed the distribution of recall results across 10 independent runs. The boxplots in Figure 7 visualize this analysis, revealing that Relation Networks exhibited the lowest stability and the highest variance in recall. For instance, in the 2-way, 10-shot configuration for the Hernia class, the model reported a recall of 28.4% ± 12.55, indicating that performance could drop as low as 10%. In contrast, MAML and FoMAML demonstrated the most consistent behavior across runs, with the lowest observed variance. Prototypical Networks followed closely in terms of stability.

## 6. Discussions

This section discusses key performance trends observed across different *k*-shot settings, disease classes, evaluation metrics, and computational efficiency measures, as summarized in Table 3, Table 4, Table 5, Table 6 and Table 7.

The results of the backbone comparison, summarized in Table 3, underscore the critical role of the feature extractor in meta-learning, as it encodes rich representations that directly influence the model’s ability to generalize to novel classes. In our experiments, DenseNet-121 consistently achieved the highest performance when integrated with the Prototypical Network, with a recall of 68.1% ± 1.7. ResNet-50 followed closely with 67.3% ± 2.1, producing competitive results. In contrast, ConvNeXt-Tiny—a recent convolutional architecture inspired by transformer design—underperformed significantly, with a recall of only 60.9% ± 7.3. This suggests that, despite its promising results in large-scale benchmarks, ConvNeXt may struggle in low-data regimes typical of few-shot medical tasks. ViT-Base, the transformer-based model, also showed limited effectiveness in this setting, obtaining the lowest recall (54.3% ± 2.8), likely because it requires large amounts of training data. Although no statistically significant difference was found between DenseNet-121 and ResNet-50, DenseNet-121 is selected as the most suitable backbone for its slightly higher performance and substantially lower parameter count (6.95 M vs. 23.50 M), which reduces overfitting risk and computational cost. A plausible explanation for DenseNet’s slightly superior performance lies in its feature reuse mechanism. By concatenating feature maps from all preceding layers, DenseNet-121 creates richer and more discriminative embeddings. This is particularly advantageous for Prototypical Networks, as it allows for the formation of more stable and representative class prototypes, which is crucial for distinguishing between visually similar medical conditions.

Regarding the evaluated meta-learning models—Prototypical Network, Relation Network, MAML, and FoMAML, all implemented with a DenseNet-121 backbone—the Prototypical Network emerges as the most effective, distinguished by its simplicity, computational efficiency, and strong adaptability to few-shot classification tasks. While MAML incurs higher computational overhead due to second-order optimization, FoMAML offers a more efficient alternative by relying only on first-order gradients, achieving comparable results. Overall, the Prototypical Network exhibits the most consistent and superior performance, particularly in adapting to novel disease classes. A detailed comparison of performance across all metrics and *k*-shot configurations is provided in Table 4. Also, statistical results consistently identify Prototypical Networks as the top-performing meta-learning approach, thereby reinforcing the validity of the observed performance trends.

Table 5 presents another key finding of this study. The best class-level performance was obtained for Hernia, where MAML achieved a mean precision of 0.600 in the 2-way, 10-shot setting, representing the highest score across all configurations. Notably, Hernia is also the class with the fewest available samples, suggesting that the few-shot learning approach is particularly effective in handling data-scarce conditions. In contrast, Pneumonia proved to be the most challenging condition, consistently yielding the lowest mean recall across nearly all evaluated models.

Prototypical Networks and Relation Networks require approximately 17% fewer FLOPs for inference and about 25% fewer FLOPs for training compared to optimization-based approaches (Table 6). This reduction stems from the fact that metric-based methods perform classification by computing distances in an embedding space without iterative gradient updates within each episode. In contrast, optimization-based methods such as MAML and FoMAML require inner-loop gradient computations for task-specific adaptation, which substantially increases the number of operations, particularly during meta-training. This computational gap is also reflected in execution times (Table 7), where metric-based approaches complete meta-training episodes significantly faster than their optimization-based counterparts. Although meta-testing times are lower for all methods, MAML and FoMAML remain considerably slower than ProtoNet and RelationNet due to the additional task-specific adaptation step performed during evaluation.

These findings directly address the objectives of this study: evaluating the influence of backbone architectures and comparing representative meta-learning strategies in few-shot chest X-ray classification.

When situating our work within the context of closely related studies, two contributions stand out for direct comparison. The first is the work by Paul et al. [35], which combined a DenseNet-121 architecture as a feature extractor with an ensemble of 15 autoencoders. They evaluated their approach on the ChestX-ray14 and Open-i datasets, testing various combinations of novel and base classes using a 3-way, 5-shot setting, whereas our study constructs meta-tasks using a single dataset. Although this prevents us from making a direct comparison, it offers useful insights into their method’s performance relative to our best model. The authors reported precision values of 0.35 ± 0.07 for hernia, 0.44 ± 0.03 for pneumonia, and 0.46 ± 0.02 for edema. In comparison, our strategy achieves precision scores of 0.545 ± 0.026 for hernia, 0.502 ± 0.001 for pneumonia, and 0.527 ± 0.011 for edema. These results highlight the competitiveness of our method while utilizing a simpler model configuration. Their approach, although innovative, is computationally intensive. Moreover, while their meta-testing protocol combines various novel and base class configurations within the same fixed setting, our experimental design applies a controlled 2-way protocol, training on the 11 most frequent diseases in the ChestX-ray14 dataset and testing exclusively on the three least frequent classes. By systematically varying the number of shots (5, 7, and 10), our analysis provides deeper insights into few-shot generalization, which is not explored in their work.

A second relevant study is that of Ouahab et al. [34], who introduced ProtoMed by augmenting a Prototypical Network with a supervised contrastive branch and evaluated Conv4, ResNet10, and DenseNet backbones. Their experiments explored 3-, 5-, and 10-shot settings, primarily focusing on accuracy. They reported a peak accuracy of 78.36% in a 2-way 10-shot configuration. However, this result is not directly comparable to ours, as their protocol included an additional class from another dataset. Furthermore, their evaluation was limited to PA-view single-label images at 224 × 224 resolution, without balancing for class distribution. In contrast, our study incorporated both PA and AP views, applied CLAHE preprocessing and SMOTE balancing, and extended the evaluation with disease-specific analysis.

Overall, our study advances beyond prior works by unifying methodological rigor with clinical relevance. Through a lightweight metric-based model and systematic few-shot evaluation, we establish a robust framework for generalization in rare thoracic disease classification. Notably, the competitive precision results obtained highlight the model’s applicability as a decision-support system, complementing clinical judgment by reducing diagnostic uncertainty, especially in data-scarce scenarios where timely guidance is essential.

## 7. Conclusions

This study highlights the critical role of backbone selection in few-shot medical image classification. Of the five evaluated architectures, DenseNet-121 demonstrated the highest performance when combined with a Prototypical Network, clearly surpassing ConvNeXt and ViT under data-scarce conditions. These findings indicate that densely connected CNNs remain more effective than transformer-inspired architectures in low-data medical scenarios.

When comparing meta-learning strategies, Prototypical Networks consistently achieved the best overall balance between accuracy and computational efficiency, confirming the suitability of metric-based approaches for rapid adaptation in clinical contexts. Nonetheless, MAML and FoMAML obtained higher per-class scores in Pneumonia and Hernia, indicating that gradient-based methods may be advantageous in certain scenarios despite their higher training costs.

Beyond the numerical results, this work contributes a unified evaluation protocol that integrates contrast enhancement, data balancing with SMOTE, and systematic variation of k-shot configurations. This methodological rigor provides a more comprehensive understanding of model generalization in few-shot medical imaging.

Finally, while the experiments were limited to three rare diseases under a 2-way setting, the results demonstrate the potential of meta-learning for addressing data scarcity in thoracic disease classification. Looking forward, specific strategies can be adopted to strengthen few-shot learning in thoracic disease classification. Key approaches include the adoption of advanced data augmentation techniques (e.g., CutOut, MixUp, CutMix), the design of cross-dataset meta-learning protocols to improve robustness across heterogeneous acquisition settings, and the development of task-adaptive loss functions to enhance inter-class separability. These refinements are expected to yield more reliable performance under challenging conditions and bring few-shot learning approaches closer to clinical applicability. Future research should extend this framework to multi-label and higher-way tasks, incorporate domain-specific pretraining, and explore interpretability methods to enhance the clinical applicability of few-shot learning approaches.

## Figures and Tables

**Table 1:** Distribution of images for base and novel classes in meta-training and meta-testing, before and after SMOTE.

Class	Original Number of Images	Synthetic Samples Generated (SMOTE)	Final Number of Images
Base classes			
Cardiomegaly	1093	8454	9547
Nodule	2705	6842	9547
Emphysema	892	8655	9547
Effusion	3955	5592	9547
Atelectasis	4215	5332	9547
Mass	2139	7408	9547
Pneumothorax	2194	7353	9547
Pleural Thickening	1126	8421	9547
Fibrosis	727	8820	9547
Consolidation	1310	8237	9547
Infiltration	9547	0	9547
Total Base	29,903	75,114	105,017
Novel classes			
Edema	628	0	628
Pneumonia	322	0	322
Hernia	110	0	110
Total Novel	1060	0	1060

**Table 2:** Recall scores (%) of different backbones within Prototypical Network across 5-shot, 7-shot, and 10-shot settings.

Backbone	5-Shot	7-Shot	10-Shot
DenseNet-121	64.5 ± 2.3	66.6 ± 1.7	68.1 ± 1.7
ResNet-50	63.7 ± 2.5	65.3 ± 2.4	67.3 ± 2.1
MobileNetV2	64.6 ± 3.5	64.7 ± 3.1	64.4 ± 3.8
ViT-Base	52.6 ± 2.2	54.4 ± 2.6	54.3 ± 2.8
ConvNeXt-T	54.0 ± 3.3	55.1 ± 5.0	60.9 ± 7.3

**Table 3:** Results of different backbones within Prototypical Network, evaluated on novel disease classes under a 2-way, 10-shot setting.

Backbone	Recall (%)	Precision	F1-Score (%)
DenseNet-121	68.1 ± 1.7	0.693 ± 0.018	67.4 ± 1.6
ResNet-50	67.3 ± 2.1	0.686 ± 0.024	66.6 ± 2.1
MobileNetV2	64.4 ± 3.8	0.646 ± 0.037	63.5 ± 3.9
ViT-Base	54.3 ± 2.8	0.548 ± 0.030	52.8 ± 3.0
ConvNeXt-T	60.9 ± 7.3	0.615 ± 0.082	58.9 ± 9.0

**Table 4:** Performance of meta-learning models evaluated on novel classes under 2-way classification with varying *k*-shot settings.

*k*-Shot	Model	Recall (%)	Precision	F1-Score (%)
5-shot	MAML	60.4 ± 0.6	0.626 ± 0.008	57.3 ± 0.7
	FoMAML	60.7 ± 0.3	0.628 ± 0.004	57.8 ± 0.5
	Relation Network	50.2 ± 1.8	0.430 ± 0.076	41.2 ± 4.3
	Prototypical Network	67.2 ± 1.4	0.685 ± 0.016	66.4 ± 1.4
7-shot	MAML	60.8 ± 0.7	0.630 ± 0.008	57.6 ± 0.7
	FoMAML	61.8 ± 0.2	0.641 ± 0.002	58.9 ± 0.2
	Relation Network	49.3 ± 4.4	0.416 ± 0.091	40.5 ± 5.8
	Prototypical Network	67.2 ± 1.5	0.684 ± 0.016	66.4 ± 1.6
10-shot	MAML	61.9 ± 0.8	0.644 ± 0.010	59.0 ± 0.8
	FoMAML	62.4 ± 0.5	0.648 ± 0.005	59.6 ± 0.6
	Relation Network	50.7 ± 6.9	0.481 ± 0.101	45.2 ± 8.2
	Prototypical Network	68.1 ± 1.7	0.693 ± 0.018	67.4 ± 1.6

**Table 5:** Recall (%), Precision, and F1-score (%) per class across a 2-way, 10-shot configuration. Best results for each metric and class are in bold.

Model	Pneumonia			Hernia			Edema		
	Recall	Precision	F1	Recall	Precision	F1	Recall	Precision	F1
MAML	**34.0 ± 1.4**	**0.532 ± 0.010**	**39.5 ± 1.4**	**46.6 ± 1.6**	**0.600 ± 0.011**	**50.8 ± 1.4**	29.8 ± 1.3	0.510 ± 0.008	35.8 ± 1.2
FOMAML	33.9 ± 0.7	0.528 ± 0.006	**39.5 ± 0.6**	46.3 ± 1.0	0.599 ± 0.006	50.6 ± 0.9	30.4 ± 0.8	0.513 ± 0.004	36.4 ± 0.7
Relation Net	30.0 ± 7.3	0.499 ± 0.032	35.1 ± 6.1	28.4 ± 12.5	0.462 ± 0.131	32.4 ± 13.0	33.4 ± 14.3	**0.533 ± 0.061**	38.5 ± 12.6
Proto Net	28.8 ± 0.6	0.502 ± 0.001	35.7 ± 0.5	41.2 ± 5.5	0.545 ± 0.026	46.2 ± 4.6	**38.7 ± 2.7**	0.527 ± 0.011	**44.0 ± 2.2**

**Table 6:** Per-episode and total FLOPs for training and inference computations, measured during the meta-training phase for each evaluated model.

Model	FLOPs/Episode		Total FLOPs	
	Training	Inference	Training	Inference
ProtoNet	111 GFLOPs	37 GFLOPs	111 TFLOPs	37 TFLOPs
RelationNet	109.30 GFLOPs	36.43 GFLOPs	109.30 TFLOPs	36.43 TFLOPs
MAML	327.48 GLOPs	218.32 GFLOPs	327.48 TLOPs	218.32 TFLOPs
FoMAML	272.90 GFLOPs	218.32 GFLOPs	272.90 TFLOPs	218.32 TFLOPs

**Table 7:** Average execution time per episode and total time during meta-training and meta-testing phases for each model.

Model	Phase	Time/Episode	Total Time
ProtoNet	Meta-training	0.0515 s	48.36 s
	Meta-testing	0.0309 s	29.88 s
RelationNet	Meta-training	0.0642 s	51.64 s
	Meta-testing	0.0268 s	24.47 s
MAML	Meta-training	2.60 s	2642 s
	Meta-testing	0.439 s	441 s
FoMAML	Meta-training	0.97 s	1050.61 s
	Meta-testing	0.43 s	443.34 s

## Data Availability

The implemented models used in this study can be found in the following GitHub repository: https://github.com/Chardqui/meta-learning-chest-xrays.
